# Perspectives of organizational identity in a health higher education institution: a mixed-method analysis

**DOI:** 10.1186/s12909-020-02470-1

**Published:** 2021-01-14

**Authors:** Ubirajara José Picanço de Miranda Junior, Maria Rita Carvalho Garbi Novaes, Henrique Batista Araújo Santos, João Fellipe Santos Tatsch, Rafael Sanches Ferreira, Wilton Paulo de Freitas Martins Vieira, Luís Velez Lapão

**Affiliations:** 1 Escola Superior de Ciências da Saúde (ESCS) - Fundação de Ensino e Pesquisa em Ciências da Saúde (FEPECS) - Secretaria de Estado de Saúde do Distrito Federal (SESDF), SMHN Qd. 03, Conjunto A, Bloco 1, Edifício FEPECS, Brasília, 70.710-907 DF Brazil; 2 Faculdade de Ciências da Saúde (FS), Universidade de Brasília (UnB), Campus Universitário Darcy Ribeiro, Asa Norte, Brasília, 70.910-900 DF Brazil; 3 Instituto de Higiene e Medicina Tropical (IHMT), Universidade Nova de Lisboa (UNL), Rua da Junqueira, 100, 1349-008 Lisbon, Portugal

**Keywords:** Academic performance, Health occupations schools, Organization and administration, Organizational culture, Organizational identity, Organizational objectives, Universities

## Abstract

**Background:**

Among the processes to be experienced by any organization during its establishment is the formation of an organizational identity. This process can be understood as the activity and event through which an organization becomes unique in the mind of its members. An organizational identity leads to an identification and both are directly associated with the success of an institution. This study is about a public higher education institution in health in its early years, with distinctive characteristics in the country where it is situated. In spite of having been successful in the graduation of its students it has fragile institutional bases, lack of autonomy and internal problems common to other institutions of this type. Thus, this study was conducted to understand how this institution defined itself among its own members, the elements of its identity and what justified its relative success despite its weaknesses.

**Methods:**

A mixed-method approach was used to evaluate how a representative portion of this organization identifies with it. For the qualitative study two focus groups were conducted with transcripts submitted to content analysis proposed by Bardin, culminating in results from which a Likert scale-based questionnaire was elaborated and applied to 297 subjects.

**Results:**

There were six central elements of the organizational identity made evident by the focus groups: political / ideological conflict; active teaching and learning methodologies; location / separation of campuses; time of existence; teaching career; political-administrative transformations. The quantitative analysis revealed in more detail the general impressions raised in the focus groups. Most results were able to demonstrate distinct identifications of the same identity with its exposed weaknesses.

**Conclusions:**

Lack of autonomy, administrative and structural shortcomings and ideological or political conflicts presented themselves as problems capable of destabilizing the identity of a public higher education institution. On the other hand, one way to combat such problems is through the development of the institution itself, particularly by becoming more active and useful to the community and seeking in a common interest to the higher administration agencies.

**Supplementary Information:**

The online version contains supplementary material available at 10.1186/s12909-020-02470-1.

## Background

The establishment of a Higher Education Institution (HEI) always presupposes an objective, a finality focused on the needs of a population seeking a common good. In the case of a public health education institution, this finality should be even more evident, since the training of professionals in this area has the potential to contribute directly to the improvement of socio-economic indicators in the region [1, 2].

However, the success of a health education institution in educating good professionals and making a real difference to the region where it is situated is not immediate and undergoes daily repetition of its work processes and constant adjustment to external conditions. These are natural processes to any organization (“institution” and “organization” are used interchangeably in this study) that by themselves build and shape an identity. Be it at individual, professional, social or organizational-level, identity is developed through interactions, so it is dynamic, under permanent construction [3, 4].

Organizational identity is understood as the process, activity and event through which an organization becomes unique in the minds of its members. The organization’s objective, mission, practices and values are good examples of elements that contribute to shaping its identity [1, 3, 4]. Some authors also understand organizational identity as a collective perception of the members of an organization over its central and relatively permanent characteristics that distinguish it from others [5, 6]. The formation of an identity is inevitable; thus, the real challenge is to establish what identification bonds its members will have with it. There is no identity without identification [4].

Strong identification with the organization increases cooperation between members and competition with non-members, which also determines how the organization deals with its affiliates. The feeling of identification that the individual has for his organization works as a protective force, triggering emotional processes that tend to contribute to creativity in his activities, stimulating the strengthening of this feeling [4].

For a HEI, its identity is naturally linked to its symbolic coat of arms, its values, principles and purposes, its campuses and places where it operates, the courses it offers, the forms of admission, its teaching methodologies, the curricular and extracurricular activities that determine its daily routine, in addition to other minor characteristics. All these characteristics help distinguish an educational organization as being unique [1].

However, in parallel to the formation of an organizational identity with their respective identifications derived from internal perceptions, an organizational image emerges from the perceptions external to the organization and is another determining variable in the formation and development of an institution. In unfavorable situations this image can be a destabilizing force on the organizational identity. When information from outside subjects contradicts the organizational identity, its members tend to compare and re-evaluate the way they perceive the organization. In this way, that destabilizing force can generate both positive changes or setbacks [7–9].

Regarding higher education in health, a quality education provides foundational knowledge and practical skills focused on ethical and humanistic principles for patient care. These patients ultimately assess the quality of the assistance provided by graduated professionals, reflecting the image of the organization [1–3]. The vital importance of a quality degree is evident in courses such as medicine and nursing, whose professionals often care for individuals in critical life-or-death situations.

In other words, an institution is not defined solely by external perceptions, nor solely internally by the identity it has and its fundamental characteristics. Organizational identity and image are closely correlated concepts that always co-exist, and how members define their organization is partially based on how outsiders perceive it [10].

Comprehending an institution through such concepts requires a critical study of its characteristics from the perspective of its own members, a review of its peculiarities and, more broadly, an understanding of the coherence of its historical processes leading the institution to what it is today [4]. Applying this critical study to the context of a higher education institution enables one to better understand what guides the performance of its permanent members and what will influence the performance and formation of its transitory members, the students.

This article discusses a Brazilian HEI, not only in the health sector but also an educational institution directly linked and headed by a State Health Secretariat, without representation in executive organs of Education. This is what makes it unique among Brazilian public HEIs, although it also follows the national curriculum guidelines for health education based on the tripod: Teaching, Assistance and Management [11–13].

It also stood out for being one of the first colleges to adopt active teaching methodologies in Brazil, developing educational programs focused on community assistance with the objective of graduating professionals capable of meeting the real health needs of the population in accordance with the principles and guidelines of the Brazilian Unified Health System (“SUS”) with social, ethical and human commitment [14, 15].

Despite being innovative, the link to a State Health Secretariat creates frequent conflicts of interest, causes political and administrative interference, and harms academic autonomy with direct impact on their teaching staff and employees of technical and administrative sectors.

Concerning the active teaching methodologies, the lack of comparative local parameters and the absence of previous experience by its teachers and students hindered the identification of its members with the institution itself. Moreover, it is a relatively new higher education institution, having graduated its eighth class in the year this research began, which possibly justifies the inconsistency of its image and a still little-known reputation.

Although its early years of existence have not been very favorable, curiously - since its inception the institution has always been very successful, presenting consistent results in a national evaluation system applied by the Ministry of Education to all undergraduate students in higher education courses accredited by the government.

This apparent contradiction has stimulated the comprehension of the elements that form the identity of this institution. After all, how does this health HEI define itself under the eyes of its own members? What aspects of its identity can be highlighted from this perspective? Therefore, the objective of this study was to analyze how an academic community - including its administrative components - perceives itself and its own institution, especially when its name is not well-known and does not inspire meaning among non-members.

## Methods

This is a mixed-method study with an observational, cross-sectional design carried out to evaluate how a representative portion of this organization identifies with it, whether it is a homogeneous identification among its members or whether distinct identifications prevailed, and if so, how distinct they would be.

As a quantitative research tool, a questionnaire was used to evaluate elements of identity in an expressive sample of the researched academic community. In addition, focus groups were used as a qualitative research tool to ensure data produced from questionnaires were free from induction biases and assumptions, encapsulate the most relevant issues for this academic community, and accommodate a mixed methodology approach.

Two focus groups were carried out with the main objective of stimulating free discussion on this topic so that the most relevant aspects within the comprehension of organizational identity could be identified.

### Setting

This study was conducted in the capital city of Brazil, Brasilia, at a public health HEI founded in 2001, the Escola Superior de Ciências da Saúde (ESCS - Higher School of Health Sciences). Since its inception, the School offers medical undergraduate course, medical and multidisciplinary residency programs, research projects and university extension. Since 2008 it has offered nursing undergraduate course on a secondary campus in addition to new postgraduate activities including master’s degrees *strictu* sensu since 2012 and interinstitutional doctorates since 2016.

It is an educational institution maintained by an autarchy linked to the Secretaria de Estado de Saúde do Distrito Federal (SES-DF - State Secretariat of Health of the Federal District) and the Fundação de Ensino e Pesquisa em Ciências da Saúde (FEPECS - Foundation for Teaching and Research in Health Sciences). Its teaching staff is initially selected by public tender for SES-DF and later, by another specific selection process for ESCS. The student body is admitted annually by national selection process, being eighty students per course.

In its professional formation practice, ESCS adopts active teaching methodologies with the pedagogical principles of student-centered, problem-based learning (PBL) and community-oriented learning, having the teacher as a learning facilitator. Among other educational methodologies and programs it also adopts small group learning, skills and attitudes qualification, scientific initiation, problematization and Education-Services-Community Interaction.

One of the criterion for participation in this study was the subject’s period in graduation, considering that in Brazil the regular medical education lasts 6 years, the final 2 years being mandatory curricular internship. The regular graduation in nursing lasts 4 years, the last year being mandatory curricular internship.

### Participants

Managers, technical-administrative staff and all students and professors of the first and last year of the two undergraduate courses offered by ESCS - medicine and nursing - were selected as research subjects.

For the focus groups, at least one representative from each segment of the ESCS social body - including medical and nursing courses - was chosen, restricted to undergraduate representatives the first and last years of each course. The selected segments were: director of the sponsoring institution (FEPECS), rector, course and series coordinators, course secretary, and representatives from the academic center and the ESCS faculty association, in addition to at least one lecturer and two students for each first and last year of each course, selected for their academic representation in the year to which they belong, totaling 30 participants.

The questionnaires were applied to all lecturers and students from the first and last years of medical and nursing courses, administrative members of ESCS, leading to an expected total of five hundred (500) subjects (*n* = 100%). Those who refused to participate or who, in most cases were not located in their respective lecturing or work settings during the data collection period, were excluded from the study, resulting in a final sample of 297 individuals (*n* = 59,4%) which also excludes invalid submissions.

### Focus groups

Given the difficulties in gathering participants on the same date and time, it was possible to perform two focus groups lasting just over two hours, dividing the 30 research subjects into 16 and 14 per group. In order to make the discussion more homogeneous in each group, the participants were grouped according to the administrative hierarchy of the social body.

The dynamics performed in both groups were the same, starting with the presentation of the concept of organizational identity followed by the introduction of two provocative questions: “What does ESCS mean to you?” and “What makes you feel like an ESCS member?”. Each focus group ended with brief considerations.

Due to the evident difficulty in managing the dynamics with a large number of participants, the moderator of the focus groups (the author of this research) had four researchers assist in recording important discussion points, organizing a flexible schedule of speech time, and guaranteeing every person had the opportunity to contribute.

Although the two dynamics exceeded the maximum time and the optimum number of participants, the objective of performing the focus groups was accomplished. The general participant feedback was positive regarding the quality of the discussion. The richness of the discussions and the motivation for participation was attributed to the relevance of the theme by the group participants in their feedback.

The transcripts of the groups’ recordings were submitted to Bardin’s Content Analysis [16], which was organized into six categories: political / ideological conflict; usage of active methodologies; location / separation of campuses; time of existence of ESCS; teaching career; political-administrative transformations.

From the categorized content obtained from the focus groups a Likert Scale [17] questionnaire was developed to collect quantitative data for this research, which, after statistical analysis, was again organized in the six categories mentioned above.

### Questionnaires

The questionnaire (Annex 1) used as a data collection instrument consisted of 12 statements with a series of scores numbered from one to five, to which the respondent is asked to answer among the different degrees of (dis) agreement or indifference to each statement. This questionnaire incorporated the following principles:

- Initial presentation of the concepts of organizational image and identity used in this research;
- The usage of simple and straightforward statements in a single sentence and preferably with only one verb;
- Affirmative statements with provocative content to the understanding of organizational identity, considering discussion points from the focus groups;
- Each statement addressed relevant content as a result of the Bardin Content Analysis [16] applied to the transcripts of the two focus groups.

The raw quantitative data obtained were grouped according to the different sample elements of the research (study variables):

- Main Categories: lecturer, student and administrative;
- Course: medicine and nursing;
- Years: time in graduation / performance years (first and last years).

It was observed that for the category of the administrative staff and for 23 lecturers who participated in the research the performance year did not apply as they work in more than 1 year of the course. The same observation was made for three members of the administrative group. In both cases - for the analysis of the year and course categories - only those who declared themselves active in any year or undergraduate course were considered. Participants who did not complete the questionnaire were considered only in analysis of the questions answered.

The statistical software SPSS Version 18.0.0 for Windows was used to analyze the data. Statistically significant differences were identified through the application of the Chi-Square Sequential Differentiation Test. When the sample was not large enough for its application, Fisher’s Exact Test was used to calculate the Probability of Significance (p).

Cases where the probability of significance was greater than 0.05 (*p* > 0.05) in the correlation between the research variables were excluded as there were no statistically relevant differences. The target population of the quantitative part of this study was the defined reliability for this research being 95%, determining a sampling error margin of 3.63% to more or to less.

Finally, the quantitative data was grouped into the six categories obtained in the qualitative study of this research, described in association with excerpts from the transcripts of the focus groups, fully transcribed as they were expressed, eliminating orality vices. The excerpts used were chosen due to the free association of content according to the authors’ interpretation, respecting the mentioned categories.

### Ethics

The research was initiated after its approval by the Research Ethics Committee (CEP) of the Health Sciences Teaching and Research Foundation (FEPECS), which took place on July 8, 2013, with registration at the Brazil Health Research Platform under the number of Certificate of Presentation for Ethical Appreciation (CAAE): 18328513.0.0000.5553.

Each participant in the focus groups and each respondent signed a Free and Clarified Consent Form (TCLE) to be able to participate in the project, in two copies, the first being held by the participant and the second by the researchers. The anonymity of all research subjects was ensured. Before the start of each interaction, the focus group recordings were duly authorized by all participants.

## Results

### Qualitative analysis

In order to identify relevant themes for the definition of an identity for ESCS from the perspective of its own members, the two focus groups were conducted with relative freedom in order to allow for all possible issues relevant to this objective to be raised. Six main categories of subjects treated in the focus groups were identified and each category is presented below with excerpts from the transcribed content.

#### Category 1 - Political / ideological conflict

The understanding of the interferences of the SES on the ESCS took on a political character in the focus group discussions, allowing the following statements to be highlighted:

*What bothers me is not having autonomy, the dependence we have on SES. [lecturer]*

*... what I think is more precious and the worst is the school being linked to SES. [administrative]*

*Ambivalence between what can and cannot be. Is it SES? Is it education? Is it health? This makes us all suffer in here. [administrative]*

Two other statements related to the qualitative phase of the research point more precisely to the ideological conflict between SES and ESCS regarding the different vocations between an educational institution and an assistance body of the executive power.

*Heavy contradiction in a health institution inserted in education is that they do not speak the same language... [administrative]*

*Health does not understand how education works. [administrative]*

#### Category 2 - Active methodologies

The active learning methodologies applied by the school were widely mentioned in the qualitative phase of the study, being seen as a “fundamental element of its image, especially regarding the graduation of differentiated professionals” and that distinguishes them from other schools:

*… the identity is greatly transformed by the six years of formation ... I was not aware of what it is to graduate with active methodology. [student]*

*... I don't know if we train better or worse doctors, but we train different doctors, who bother, who try to generate changes. The major milestone of the school is to train different doctors. [lecturer]*

*... I have a great identification with the school, in personal terms it represented a change in trajectory, and the results that the school has achieved over the years for me have been surprising... [lecturer]*

Despite the important emphasis on the use of active methodologies, there was an interpretive conflict for both lecturers and students:

*What I see as the best is the methodology ... even though this dream has the resistance, mainly from the lecturers, which discourages. It needs a little more training for lecturers to carry out this method, to live it. [student]*

*I see the resistance to the method in the student, a great limitation of the PBL that we do ... individual theoretical presentation, with a lot of audiovisual limitation ... The knowledge sometimes stays in the superficiality of 'ah! I presented what I had to present '. [lecturer]*

#### Category 3 - Location / separation of campuses

The findings identified as relevant in the qualitative phase point to some dissatisfaction regarding the institutional fragmentation caused by the separation of the nursing and medicine campuses:

*ESCS has a sinking dream that is the interaction of (between) the two courses... [student]*

*It is difficult to talk about the identity of ESCS students, the identity of the medicine student and the identity of the nursing student are very different. [student]*

*... the nursing campus is in Samambaia, totally distant from here (Plano Piloto) and we don't have an interaction: 'We are ESCS.'... you still don't see a recognition of ESCS - nursing. [student]*

*... there are so many things in common although we stay there in Samambaia, the people here, we have the impression that they are separated, but ... the main anguish are the same. [lecturer]*

#### Category 4 – Time of Existence

Among the themes brought up for discussion in the focus groups, the institution’s time of existence resulted in different views on consolidation of an institutional identity.

*... I think the identity is under construction and I as a student am part of it. Everyone has an identity that is not defined, but in progress. [student]*

*... it is very little time ... an external identity begins to be created as people from inside go outside and begin to present themselves in this external environment. [student]*

#### Category 5 - Teaching career

During the meeting of the focus groups, it was noted that this topic had a certain exclusivity of interest among the lecturers themselves, with little participation from the other members of the ESCS academic-social body. Some lecturers’ positions stood out:

*... I had a lot of difficulty with some colleagues who didn't understand, or who understood that we had privileges ... (that) we worked less, that this was a proposal to reduce the workload. [lecturer]*

*... you have a struggle for the weights to be equal, with the valuation of the professional (lecturer), because the boss understands that you are from there (assistance) but you are released for 20 hours (for ESCS) and then ... you seem to have two bonds but in fact here at ESCS you only have one (with SES-DF) ... [lecturer]*

#### Category 6 - Political-administrative transformations

In this regard, the following statements from the focus groups show the different ways some individuals see themselves in a School undergoing constant transformation:

*... I identify a lot with ESCS because of the challenge of learning ... to deal with each other on a daily basis, with the challenge of learning and knowing the potential that the school has to provide changes, transformations, to also understand that change is inherent to us, but that we are sometimes afraid of it, but regardless of that, the process is going on and we witness it. [lecturer]*

*... the student here has always fought a lot since a long time. Historically, it is a process of struggle that the ones who carries the fights the most, the ones who give a face (to the slap), have always been the students... [student]*

*... it is very comfortable for us to stay in our comfort zone and complain and point and say this and that; ... sometimes you find yourself doing this, because this is much easier than trying to 'build together', and 'building together' is complicated, because there are a lot of people who are already calloused by a very arduous walk and that ends up demotivating in some things... [student]*

### Quantitative analysis

Of the total of 297 questionnaires analyzed, 50 (16.8%) were answered by lecturers, 228 (76.8%) by students and 19 (6.4%) by members of ESCS management and administrative staff. The sample was also grouped according to the courses in medicine and nursing, and their respective first and last years of graduation, shown in Table 1.

**Table 1:** Sample grouping

	Medicine		Nursing		Does not apply	Total
	First year	Last year	First year	Last year		
Lecturers	18	2	6	1	23	50
Students	84	45	68	31	0	228
Managing / Administrative	7		9		3	19

The analysis of the answers to the questionnaire included lecturers, students and managerial / administrative body that declared themselves members of the respective courses.

The twelve questions that made up the questionnaire were distributed among the six categories produced by the qualitative analysis of the focus groups as shown in Table 2.

**Table 2:** Questions by categories

	Questions
Category 1 - Political / ideological conflict	*“The DF Health Department recognizes the role of ESCS, but does not identify with the school”;* *“There is an ideological conflict between SES and ESCS, since the first has a vocation in assistance and not in education”.*
Category 2 - Active methodologies	*“At ESCS there is a methodological conflict in the teaching-learning process”;* *“Lecturers at ESCS did not effectively incorporate the adopted methodology”;* *“The students at ESCS did not effectively incorporate the adopted methodology”.*
Category 3 - Location / separation of campuses	*“The Samambaia campus represents ESCS as much as the Plano Piloto campus”;* *“The separation of medicine and nursing courses on two campuses makes organizational identity difficult”.*
Category 4 - Time of existence	*“ESCS’s existence has not been long enough for its identification”;* *“There are several ESCS, including one real and the other ideal”.*
Category 5 - Teaching career	*“Because there is no teaching career at the institution, the faculty does not feel like an integral part”.*
Category 6 - Political-administrative transformations	*“As for participation in the face of the political changes that are happening at ESCS, you ...”;* *“With the creation of the university, the ESCS social body will feel more strengthened in its identification with the institution”.*

The quantitative analysis of the questionnaires expanded on the general findings in the focus groups. Grouping the sample across members of the academic community, courses and year of graduation allowed us to better contrast the perception of the institution’s identity among participants, especially among students of different courses or in different periods of the time of study within the graduation.

#### Category 1 - Political / ideological conflict

On the questions relating to the political / ideological conflict present at the institution, it is worth mentioning that the levels of agreement were different between teachers and students regarding the statement: “The Health Department of the DF recognizes the role of ESCS, but does not identify with the school”, unlike the second statement about ideological conflict in which the degrees of agreement were predominantly more distinct among members of different graduation years.

It also draws attention to the higher percentage of indifference among the first year members in both courses for both statements and among students compared to teachers. See details in Tables 3 and 4.

**Table 3:** The Health Department of the DF recognizes the role of ESCS, but does not identify with the school

	Totally Disagree	Disagree	Indifferent	Agree	Totally Agree	Total
Lecturers	1 (2%)	10 (20%)	1 (2%)	25 (50%)	13 (26%)	50 (100%)
Students	18 (8.0%)	73 (32.6%)	36 (16.1%)	68 (30.4%)	29 (12.9%)	224 (100%)

*p*< 0.001 – There was no statistical significance in the comparison with managers / administrators

**Table 4:** There is an ideological conflict between SES and ESCS, since the first has a vocation in assistance and not in education

	Totally Disagree	Disagree	Indifferent	Agree	Totally Agree	Total
First	16 (9.5%)	57 (33.9%)	48 (28.6%)	40 (23.8%)	7 (4.2%)	168 (100%)
Last	3 (3.8%)	22 (27.8%)	6 (7.6%)	33 (41.8%)	15 (19.0%)	79 (100%)

*p<* 0.001

#### Category 2 - Active methodologies

As for the presence of methodological conflict in the teaching-learning process at ESCS, in general, the individuals surveyed expressed a balance between agreement and disagreement.

Regarding the fact that lecturers did not effectively incorporate the methodology adopted by the School, Table 5 shows that a larger portion of teachers agreed with this statement when compared to the level of agreement of the students. As for the statement that “ESCS students did not effectively incorporate the adopted methodology”, there were clear differences between lecturers and students (Table 6).

**Table 5:** ESCS lecturers did not effectively incorporate the adopted methodology

	Totally Disagree	Disagree	Indifferent	Agree	Totally Agree	Total
Lecturers	1 (2.0%)	23 (47.0%)	5 (10.2%)	14 (28.6%)	6 (12.2%)	49 (100%)
Students	65 (28.8%)	93 (41.2%)	19 (8.4%)	41 (18.1%)	8 (3.5%)	226 (100%)
First	64 (37.0%)	79 (45.7%)	16 (9.2%)	14 (8.1%)	0 (0%)	173 (100%)
Last	2 (2.5%)	29 (36.7%)	5 (6.3%)	33 (41.8%)	10 (12.7%)	79 (100%)

*p<* 0.001

**Table 6:** ESCS students did not effectively incorporate the methodology adopted

	Totally Disagree	Disagree	Indifferent	Agree	Totally Agree	Total
Lecturers	3 (6%)	30 (60%)	1 (2%)	14 (28%)	2 (4%)	50 (100%)
Students	49 (21.8%)	123 (54.7%)	19 (8.4%)	29 (12.9%)	5 (2.2%)	225 (100%)
First	38 (22.0%)	101 (58.3%)	15 (8.7%)	15 (8.7%)	4 (2.3%)	173 (100%)
Last	13 (16.5%)	41 (51.9%)	5 (6.3%)	19 (24.0%)	1 (1.3%)	79 (100%)

*p*=0.007 when comparing members of the academic community

*p*=0.021 in the comparison between years

It was an expressive perception of the members of the last years of medicine and nursing courses that there was no due “incorporation” of the active methodologies adopted by the teachers or the students (Tables 5 and 6).

#### Category 3 - Location / separation of campuses

Table 7 shows that for the statement: “The Samambaia (nursing) campus represents ESCS as much as the Plano Piloto (medicine) campus” the share of nursing representatives who totally disagreed with the statement (47.0%) surpassed that of medicine by more than four times (11.2%).

**Table 7:** The Samambaia campus represents ESCS as much as the Plano Piloto campus

	Totally Disagree	Disagree	Indifferent	Agree	Totally Agree	Total
Medicine	16 (11.2%)	36 (25.1%)	37 (25.9%)	38 (26.6%)	16 (11.2%)	143 (100%)
Nursing	70 (47.0%)	37 (24.8%)	5 (3.4%)	17 (11.4%)	20 (13.4%)	149 (100%)
First	43 (24.7%)	45 (25.9%)	29 (16.7%)	34 (19.5%)	23 (13.2%)	174 (100%)
Last	35 (44.3%)	18 (22.8%)	11 (13.9%)	10 (12.7%)	5 (6.3%)	79 (100%)

*p<* 0.001 when comparing courses

*p*=0.026 when comparing years

Table 8 depicts this same trend with the predominant agreement that “The separation of medicine and nursing courses on two campuses makes organizational identity difficult”, especially among nursing representatives.

**Table 8:** The separation of medicine and nursing courses on two campuses makes organizational identity difficult

	Totally Disagree	Disagree	Indifferent	Agree	Totally Agree	Total
Medicine	14 (9.9%)	31 (21.8%)	18 (12.7%)	42 (29.6%)	37 (26.0%)	142 (100%)
Nursing	5 (3.4%)	13 (8.7%)	8 (5.4%)	50 (33.5%)	73 (49.0%)	149 (100%)
First	17 (9.8%)	33 (19.0%)	21 (12.1%)	57 (32.9%)	45 (26.0%)	173 (100%)
Last	0 (0%)	5 (6.3%)	3 (3.8%)	23 (29.1%)	48 (60.8%)	79 (100%)

*p<* 0.001

#### Category 4 - Time of existence

The academic community understands the statement “ESCS’s existence has not been sufficient for its identification” in a similar and uniform way, with no statistically significant differences in any comparison, either between grades, courses or representatives of the institution’s social body.

Similarly, for the statement: “There are several ESCS, including one real and the other ideal”, there was only a statistically significant difference in the comparison of agreements between lecturers and administrative members, with a clear polarization of opinions between the latter group as shown in Table 9.

**Table 9:** There are several ESCS, including one real and the other ideal

	Totally Disagree	Disagree	Indifferent	Agree	Totally Agree	Total
Lecturers	2 (4%)	6 (12%)	6 (12%)	28 (56%)	8 (16%)	50 (100%)
Technical-administrative	0 (0%)	9 (47.3%)	1 (5.3%)	8 (42.1%)	1 (5.3%)	19 (100%)
First	9 (5.2%)	57 (32.9%)	37 (21.4%)	63 (36.4%)	7 (4.1%)	173 (100%)
Last	1 (1.3%)	5 (6.4%)	3 (3.9%)	43 (55.1%)	26 (33.3%)	78 (100%)

*p*=0.035 when comparing members of the academic community

*p<* 0.001 when comparing years

#### Category 5 - Teaching career

The statement: “As there is no teaching career at the institution, the faculty does not feel like an integral part” raised very heterogeneous opinions among ESCS’s social staff as shown in Table 10. Only lecturers showed a tendency to agree with it.

**Table 10:** As there is no teaching career at the institution, the faculty does not feel like an integral part

	Totally Disagree	Disagree	Indifferent	Agree	Totally Agree	Total
Lecturers	5 (10%)	15 (30%)	1 (2%)	21 (42%)	8 (16%)	50 (100%)
Students	47 (20.8%)	89 (39.4%)	48 (21.2%)	30 (13.3%)	12 (5.3%)	226 (100%)
Technical-administrative	3 (16.7%)	6 (33.3%)	4 (22.2%)	5 (27.8%)	0 (0%)	18 (100%)
First	45 (25.9%)	76 (43.7%)	32 (18.4%)	15 (8.6%)	6 (3.4%)	174 (100%)
Last	2 (2.5%)	24 (30.4%)	16 (20.3%)	26 (32.9%)	11 (13.9%)	79 (100%)

*p*< 0.001 in the comparison between lecturers and students

*p*=0.023 in the comparison between lecturers and technical-administrative

*p<* 0.001 when comparing years

#### Category 6 - Political-administrative transformations

The active participation of ESCS members in the face of the constant changes experienced by the institution was evaluated according to different degrees of involvement based on the statement: “As for participation in the face of political changes that are happening at ESCS, you...”. The possible responses were graded according to the Likert scale [17], ranging from “considering it important and participating in all meetings and discussions” to “not considering it important”. The results are presented in detail in Table 11.

**Table 11:** As for participation in the face of political changes that are happening at ESCS, you ..

	Considers it important and participates in discussions	Considers it important, but does not participate in discussions	Considers it important, but not very productive	Has no opinion on the topic	Does not consider it important	Total
Lecturers	11 (22.4%)	22 (44.9%)	12 (24.5%)	4 (8.2%)	0 (0%)	49 (100%)
Students	28 (12.4%)	133 (59.1%)	13 (5.8%)	51 (22.7%)	0 (0%)	225 (100%)
Technical-administrative	1 (5.3%)	10 (52.6%)	4 (21.0%)	3 (15.8%)	1 (5,3%)	19 (100%)
First	23 (13.4%)	102 (59.3%)	6 (3.5%)	41 (23.8%)	0 (0%)	172 (100%)
Last	7 (8.9%)	47 (59.5%)	11 (13.9%)	14 (17.7%)	0 (0%)	79 (100%)

*p<* 0.001 when comparing members of the academic community

*p*=0.021 when comparing years

Table 12 shows the varying degrees of agreement regarding the statement “With the creation of the university, ESCS’s social body will feel more strengthened in its identification with the institution”. Probably because it was a subject little publicized in that period, the percentages of “indifferent” responses were high. Finally, nursing representatives were considerably more optimistic about the change.

**Table 12:** With the creation of the university, the ESCS social body will feel more strengthened in its identification with the institution

	Totally Disagree	Disagree	Indifferent	Agree	Totally Agree	Total
Medicine	19 (13.3%)	27 (18.9%)	31 (21.7%)	55 (38.4%)	11 (7.7%)	143 (100%)
Nursing	2 (1.3%)	10 (6.8%)	40 (27.0%)	63 (42.6%)	33 (22.3%)	148 (100%)
First	10 (5.8%)	14 (8.1%)	48 (27.7%)	73 (42.2%)	28 (16.2%)	173 (100%)
Last	10 (12.6%)	15 (19.0%)	18 (22.8%)	29 (36.7%)	7 (8.9%)	79 (100%)

*p<* 0.001 when comparing courses

*p*=0.018 when comparing years

Quantitative data is described in full and in detail in Additional file 2.

## Discussion

The focus groups brought to light issues that were considerably relevant to the organizational identity of the ESCS. According to the interviewees themselves, the discussions stimulated by the group dynamics made it possible to go deeper into the subject, revealing potential means to keep the identity of this institution stable or to destabilize it.

Through the analysis of the questionnaire data, different interpretations about the organizational identity among the studied groups were expected. It was also expected that opinions would differ between individuals in the same course, but with different program years. These assumptions were in part confirmed and reinforce the thesis of the weak organizational identity of ESCS, disagreements of identification among its members and consequent interference in the productive processes of this organization.

So, by drawing an analytical overview of the results obtained, also compared to the first suppositions of this study, it was possible to understand how this academic community identifies itself and how it perceives the institution of which it is part. This analysis was organized in two large groups, one to deal with the political and ideological aspects of the organizational identity of ESCS and another to discuss its operational and structural aspects.

### Political and ideological conflict and political-administrative implications

The management frameworks in the healthcare area are primarily care-oriented. The fact that ESCS is a Higher Education Institution related to the Secretary of State of Health of the Federal District allows for political and ideological conflict between the interpretation of the inherent function of health care by SES-DF and the vocational teaching function of the School [18–20].

The discussion about this duality was largely a part of the dynamics carried out in the focus groups. The understanding that health (from the assistance point of view) and education are distinct and divergent was prevailing. In practice, the financial resources that reach ESCS come mostly from SES, which has no mechanisms or practical means to accurately assess the financial needs of health education. It is similar to what occurs with the human resources of ESCS, which are also almost all provided by SES-DF.

In this regard, the quantitative results show that only the last year’s graduates tend to associate the ideological conflict between SES and ESCS with their respective vocations, health assistance and education. The impossibility of directly managing its own resources compromises not only its administration, but also harms its autonomy. For lack of self-government, ESCS is constantly subject to generally imposed political and administrative interference, requiring its members to actively participate in movements to defend institutional rights or interests.

Autonomy, among other attributes, is traditionally regarded as a feature of institutions with strong organizational identities. With it also rises a sense of organizational responsibility, distributing it not only among managers but to all members of the organization. Each component of the system then becomes equally important for institutional development [21].

The focus groups provided valuable insight into this matter, highlighting the difficulties imposed by the changes, the leading role of students in the face of institutional struggles, and the need for cohesion in a discontented academic community. The quantitative data were also interesting in regards to the participation of each surveyed in the face of the political changes taking place in ESCS. Almost 80% of respondents pointed out “political changes” as important, however among these the group that “does not participate in the discussions” prevailed. This same response was prevalent in all categories of the ESCS staff, even among the years.

Neave [22, 23] introduced two contrasting notions of the private and public definition of university autonomy, which can be applied to ESCS. The private definition of university autonomy is anchored in the concepts of academic freedom and self-government. It concerns the right of the university body to determine the nature of its academic work (academic freedom) and refers to the purposes and functions for which the universities themselves are responsible (self-governance).

The public definition of university autonomy refers to the purposes and functions of higher education institutions (HEIs) determined by stakeholders, including political groups, other academic communities, employers’ organizations and trade unions, as well as society itself. A fundamental aspect of the public definition is the influence of external stakeholders on the university [22, 23].

The reality of the ESCS in this sense is the predominance of the interests of political groups over the interests of its academic community in addition to the institution’s own legal limitation on its self-governance. Consequently, its members experience the constant state of institutional conflict, highlighted by this research. The data obtained in this study exposes institutional weaknesses of unstable management and frequent political and administrative interventions, divergent from the interests of their own community.

This scenario reinforces the assumption that the organizational identity of the ESCS remains “weak” to date. According to Stensaker, weak identities are open to several “potential interpretations”, contestation and tensions, and may incite various power struggles and influence over the future profile of the institution. In universities with strong and distinct identities such disagreements may be rarer, thus reducing the need to adjust and deal with new and challenging situations [24].

Furthermore, referring to Neave’s public definition of university autonomy [22, 23], the limited influence of society in this institution, as a stakeholder, results from still being little known to the community, which is gradually changing year by year as ESCS educates its students and as it engages in changes in health services for the population.

### Organizational structuring (location of campuses, active methodologies, creation time and lecturing career)

The time of existence of ESCS has been mentioned in the focus groups as one of the determining elements for its development as an institution. There was a consensus that, within a time logic, internally the organizational identity is under construction in each of its members and externally ESCS has become increasingly known, shaping its image. In this process its identity tend to vary as new perceptions of itself and its role in the context to which it is inserted, especially if it still maintains a fragile organizational identity [10, 24].

In the questionnaires, a small majority disagreed that the short time of existence of ESCS was insufficient for the design of an identification, what also does not diverge from the opinion prevailing in the focus groups that the formation of the ESCS’ identity is a process in course. Although just over 60% of the total sample believes that the lifetime of the ESCS has not prevented it from having some identity, this same series-divided sample shows that almost 90% of the final years of graduation agree that an ideal ESCS has not yet been achieved. It is a new and innovative institution that has given good results from the outset, a characteristic of institutions with a firm organizational identity [24, 25], but its internal reality still does not inspire security to its own members. In other words, it is success achieved on fragile foundations.

One of these bases, pointed out by this research, is the absence of a career for the institution’s teachers. As already mentioned, the teaching at ESCS is characterized by the health professional of SES-DF who develops his educational activities integrated to his workload in the assistance. However, since the teachers are exclusively assigned by SES and there is no legal provision for this career in this Secretariat, there is no guarantee of their permanence in the teaching position, directly damaging the bond between this professional with ESCS and finally, their identification with the School [26].

In the focus groups this subject was discussed almost exclusively among the teachers present; in the questionnaires the teachers were the only majority who agreed that the absence of a career makes them not feel part of the institution. According to the quantitative results, perhaps because of a better understanding of this reality over the years, only those in the final years of the courses came close to this same perception. The difficulty of understanding this demand from teachers is understandable because this is a practical and professional problem, but although it is not noticed by the majority of ESCS’ social body, the results present the existence of the problem, potentially damaging to the institution.

Going a little further than teaching, the active methodologies applied at ESCS were also approached as one of the defining elements of its identity. One idea not refuted in the focus groups and demonstrated in the qualitative results is that the methodologies applied not only make it a differentiated institution, but also justify much of its educational success. On the other hand, the discussions also revealed that neither teachers nor students had effectively incorporated the methodologies.

Most of these teachers were trained in traditional methodologies, then the effective implementation of the method occurs through an initial training and quality continuing education program. Like the lecturers, most students have never had experience with active methodologies, making the implementation of the method a responsibility of all involved [19, 26].

In this sense, some self-criticism can be observed when, compared to the level of agreement of the students, the teachers showed a greater degree of agreement about not having effectively incorporated the methodology adopted by the School. As for the students not having effectively incorporated the methodology adopted and in opposition to what had been discussed in the focus groups, disagreement predominated for both teachers and students.

Finally, the focal group participants reported the fragmentation of ESCS into two distinct campuses as one of the cardinal factors in the weakening of organizational identity. Initially, one of ESCS’s institutional projects was the integration of medical and nursing students formed by a different methodology, aiming at strengthening interdisciplinarity and building a holistic view of health care [27].

The results of the questionnaires showed that there is a difference in the representativeness of the degrees offered by ESCS and interference in the establishment of an identity for the School considering the separation of campuses was predominant. Although the representatives of both courses had predominantly similar opinions, nursing showed a much higher dissatisfaction.

The relevance of this matter becomes evident when 89.9% of representatives from the last years of both courses agreed to some degree that the separation of campuses makes it difficult to establish an organizational identity, which is the highest percentage of common opinions of all quantitative research.

The data collection for this work took place between the end of 2013 and the beginning of 2014, but until 2019 some reflections and (re) considerations were needed to better understand the data collected. During this period ESCS experienced new self-governance practices, implemented new educational programs and partnerships with other educational institutions and faced new conflicts of interest. Experiencing these new processes enhanced our understanding of an organizational identity in formation.

We noted that the main cohesive force capable of mitigating factors understood in this study as destabilizing to an organizational identity comes from the institution’s own development processes, especially if they are properly directed. For example, when a health HEI takes an active role in local work processes and is approved by the population who uses these services, the HEI reinforces its public utility, improves its visibility to stakeholders and strengthens its relationship with managers, which tends to ease possible ideological and political conflicts and reduce interference in its self-governance.

### Limitations of the study

The analysis of the questionnaire grouped lecturers, students and managerial / administrative body that declared themselves members of medicine or nursing. The same occurred with lecturers who declared themselves part of the framework of the first or last years of each course, which were considered with the ones from students. It is acknowledged above all in this last case, that the lack of distinction between lecturers and students impaired part of the analysis by confusing the different times of admission to the School of the students with the series of activities of the lecturers, which evidently has very different meanings.

The question regarding Table 12, “With the creation of the university, the ESCS social body will feel more strengthened in its identification with the institution”, had little or no use in discussion of the results obtained because its meaning was largely restricted to the time of data collection.

Still concerning the quantitative study, the distinction of opinions between medical and nursing courses could have been clearer if the quantitative data by series had jointly distinguished the courses.

## Conclusions

The parallel concepts of institutional image and identity propose a new way to understand an organization. Interdependent and partly derived from the point of view of its own members, the organizational identity of a HEI in health was what this research sought to understand to identify its qualities, deficiencies and potentials.

Applied to the context of the health HEI studied in this research, this model of organizational analysis helps understand how permanent and transitional members link to and perceive their educational institution from a political-administrative point of view, and finally helps identify common interests between stakeholders and the university body in health education. With greater unification of its identity among members, ESCS could play a clearer role in society with more autonomy, inspire its own members, be recognized by outsiders, and become a strong, lasting and successful institution.

Thus, the analysis of the data obtained and the possible reflections so far lead us to conclude that despite not yet having a well-established organizational identity, ESCS is moving towards it. Its components are becoming increasingly aware of its weaknesses, strengths and peculiarities, the School has not interrupted its development processes and is increasingly closer to the population.

For an institution, whether it is academic or not, unplanned changes usually involve some resistance. Going through them in a constructive and natural way may require an institutional identification with innovation, aligned to the identity of the organization. In a public institution such as ESCS, these processes may broaden teaching perspectives, add new technical and cultural values to the humanistic model of professional training, and resiliently evolve jointly with the local public health system.

## Supplementary Information

**Additional file 1 Questionnaire - Organizational Identity Project** was the quantitative instrument of this research, a Likert Scale based questionnaire.

**Additional file 2 Statistical Analysis - Organizational Identity Project** contains all the collected quantitative data already organized by categories and their respective levels of significance.

## Abbreviations

DF: Federal District
ESCS: Higher School of Health Sciences
ETESB: Technical School of Health of Brasilia
FEPECS: Health Sciences Teaching and Research Foundation
HEI: Higher Education Institution
PBL: Problem-Based Learning
SES: Secretary of State of Health
SES-DF: Secretary of State of Health of the Federal District
SUS: Brazilian Unified Health System
WHO: World Health Organization
