# Supplementary material for: Perspectives of organizational identity in a health higher education institution: a mixed-method analysis
Source: BMC Med Educ. 2021 Jan 14;21:51. doi: 10.1186/s12909-020-02470-1 (PMC7807811; doi:10.1186/s12909-020-02470-1)
Supplement: Supplementary file 1 — Additional file 1 Questionnaire - Organizational Identity Project was the quantitative instrument of this research, a Likert Scale based questionnaire. [file 12909_2020_2470_MOESM1_ESM.pdf]

Research questionnaire: **"Escola Superior de Ciências da Saúde: interpretations of organizational identity"**

Course: ( ) Medicine ( ) Nursing

Category:

a) ( ) LECTURER b) ( ) TECHNICAL ADMINISTRATIVE

Time working at ESCS:

( )Less than 1 year ( )1 to 2 years ( )3 to 5 years ( )5 to 8 years ( )More than 8 years

c) ( ) Students

Year: ( )1st ( )Last

Organizational identity is the collective understanding of the members of an organization about its central and relatively permanent characteristics. When effectively established, it is a key element in achieving and maintaining the success of the institution, even influencing the construction of a solid, mature and well-described image as to what defines it.

1. From this concept, how do you describe ESCS organizational identity?

2. What makes you feel like a member of ESCS?

3. As for participation in the face of political changes that are happening at ESCS:

( ) I consider it important and participate in ESCS meetings and discussions;
( ) I consider it important, but do not participate in ESCS meetings and discussions;
( ) I consider it important, but not very productive;
( ) I have no opinion on the topic;
( ) I do not consider it important.

For subsequent questions, a structured response model will be used, with only **one alternative per question to be answered.**

4. The DF Department of Health recognizes the role of ESCS, but does not identify with the school.

( ) totally disagree ( ) disagree ( ) indifferent ( ) agree( ) totally agree

5. There is an ideological conflict between SES and ESCS, because the former has a vocation in assistance and not in education.

( ) totally disagree ( ) disagree ( ) indifferent ( ) agree( ) totally agree

6. At ESCS there is a methodological conflict in the teaching-learning process.

( ) totally disagree ( ) disagree ( ) indifferent ( ) agree( ) totally agree

7. The Samambaia campus represents ESCS as much as the Plano Piloto campus.

( ) totally disagree ( ) disagree ( ) indifferent ( ) agree( ) totally agree

8. The separation of medicine and nursing courses on two campuses makes organizational identity difficult.

( ) totally disagree ( ) disagree ( ) indifferent ( ) agree( ) totally agree

9. The existence of ESCS has not been sufficient for its identification.

( ) totally disagree ( ) disagree ( ) indifferent ( ) agree( ) totally agree

10. With the creation of “Unisus”, the ESCS social body will feel more strengthened in its identification with the institution.

( ) totally disagree ( ) disagree ( ) indifferent ( ) agree( ) totally agree

11. Lecturers at ESCS did not effectively incorporate the methodology adopted.

( ) totally disagree ( ) disagree ( ) indifferent ( ) agree( ) totally agree

12. ESCS students did not effectively incorporate the adopted methodology.

( ) totally disagree ( ) disagree ( ) indifferent ( ) agree( ) totally agree

13. There are several ESCS, including one real and the other ideal.

( ) totally disagree ( ) disagree ( ) indifferent ( ) agree( ) totally agree

14. As there is no teaching career at the institution, the faculty does not feel as an integral part.

( ) totally disagree ( ) disagree ( ) indifferent ( ) agree( ) totally agree
