# Supplementary material for: Perspectives of organizational identity in a health higher education institution: a mixed-method analysis
Source: BMC Med Educ. 2021 Jan 14;21:51. doi: 10.1186/s12909-020-02470-1 (PMC7807811; doi:10.1186/s12909-020-02470-1)
Supplement: Supplementary file 2 — Additional file 2 Statistical Analysis - Organizational Identity Project contains all the collected quantitative data already organized by categories and their respective levels of significance. [file 12909_2020_2470_MOESM2_ESM.pdf]

Organizational Identity Project - Guide for Statistical Analysis

1. **Research question:** What is the interpretation of the organizational identity that the academic staff of ESCS has of this institution?
2. **Study variables:**
    a. Course
    b. Lecturers
    c. Students
    d. Managers
    e. Administrative Staff

Opinion of all members in general (frequency), citing divergence of opinions between groups when ***p* is** <0.05 (chi-square test and Fisher's exact test were made).

Whenever p is> 0.05, there was no statistically significant difference.

3. **Planning and sampling:**
    a. Qualitative and quantitative questionnaire
    b. Focal groups
4. **Data collection:** not randomized
5. **Data preparation:** database in SPSS
6. **Statistical Analysis**

Statistical analysis

Sample Description

Sample calculation: 184 individuals (36.8%)

Sample obtained: 297 individuals (heterogeneous)

	Lecturers	Students	Technical-Adm.	Total
Medicine	23	115	7	145
Nursing	27	113	9	149
Did not answer	0	0	3	3
Total	50	228	19	297

	Lecturers	Students	Total
First Year	24 (18 Med + 6 Nurs)	152 (84 Med + 68 Nurs)	176
Last Year	3 (2 Med + 1 Nurs)	76 (45 Med + 31 Nurs)	79
Did not answer	23 (3 Med + 20 Nurs)	0	23
Total	50	228	278

Final sample: 297 individuals (n=59.4%)

Results

Questions from 3-14

3 - As for participation in the face of political changes that are happening at ESCS:

A) Absolute and Relative Frequencies

	Considers it important and participates in discussions	Considers it important, but does not participate in discussions	Considers it important, but not very productive	Has no opinion on the topic	Does not consider it important	Total
Frequency	40 (13.8%)	163 (56.2%)	28 (9.7%)	58 (20.0%)	1 (0.3%)	290 (100%)

B) Course: p=0.082 (Fisher's exact test)

C) Categories: p<0.001 (Fisher's exact test)

	Considers it important and participates in discussions	Considers it important, but does not participate in discussions	Considers it important, but not very productive	Has no opinion on the topic	Does not consider it important	Total
Lecturers	11 (22.4%)	22 (44.9%)	12 (24.5%)	4 (8.2%)	0 (0%)	49 (100%)
Students	1 (5.3%)	10 (52.6%)	4 (21.1%)	3 (15.8%)	1 (5.3%)	19 (100%)
Technical-Adm.	28 (12.4%)	133 (59.1%)	13 (5.8%)	51 (22.7%)	0 (0%)	225 (100%)

D) Years: p=0.021 (Fisher's exact test)

	Considers it important and participates in discussions	Considers it important, but does not participate in discussions	Considers it important, but not very productive	Has no opinion on the topic	Does not consider it important	Total
First	23 (13.4%)	102 (59.3%)	6 (3.5%)	41 (23.8%)	0 (0%)	172 (100%)
Last	7 (8.9%)	47 (59.5%)	11 (13.9%)	14 (17.7%)	0 (0%)	79 (100%)

4 – The DF Department of Health recognizes the role of ESCS, but does not identify with the school

A) Absolute and Relative Frequencies

	Totally disagree	Disagree	Indifferent	Agree	Totally Agree	Total
Frequency	19 (6.6%)	86 (29.7%)	39 (13.4%)	101 (34.8%)	45 (15.5%)	290 (100%)

B) Course: $p=0.024$ (Chi-square)

	Totally disagree	Disagree	Indifferent	Agree	Totally Agree	Total
Medicine	11 (7.7%)	36 (25.4%)	15 (10.6%)	49 (34.5%)	31 (21.8%)	142 (100%)
Nursing	8 (5.4%)	50 (33.8%)	24 (16.2%)	52 (35.1%)	14 (9.5%)	148 (100%)

C) Categories: $p<0.001$ (Fisher's exact test) – there was no statistical significance in the comparison with technical administrative.

	Totally disagree	Disagree	Indifferent	Agree	Totally Agree	Total
Lecturers	1 (2.0%)	10 (20.0%)	1 (2.0%)	25 (50.0%)	13 (26.0%)	50 (100%)
Students	18 (8.0%)	73 (32.6%)	36 (16.1%)	68 (30.4%)	29 (12.9%)	224 (100%)

D) Years: $p<0.001$ (Chi-Square)

	Totally disagree	Disagree	Indifferent	Agree	Totally Agree	Total
First	16 (9.3%)	61 (35.5%)	32 (18.6%)	45 (26.2%)	18 (10.5%)	172 (100%)
Last	3 (3.8%)	19 (24.1%)	5 (6.3%)	36 (45.6%)	16 (20.3%)	79 (100%)

5 - There is an ideological conflict between SES and ESCS, because the former has a vocation in assistance and not in education

A) Absolute and Relative Frequencies

	Totally disagree	Disagree	Indifferent	Agree	Totally Agree	Total
Frequency	22 (7.7%)	86 (30.1%)	55 (19.2%)	89 (31.1%)	34 (11.9%)	286 (100%)

B) Course: $p=0.026$ (Chi-Square)

	Totally disagree	Disagree	Indifferent	Agree	Totally Agree	Total
Medicine	7 (4.9%)	41 (28.9%)	27 (19.0%)	42 (29.6%)	25 (17.6%)	142 (100%)
Nursing	15 (10.4%)	45 (31.3%)	28 (19.4%)	47 (32.6%)	9 (6.3%)	144 (100%)

C) Categories:

Lecturers X Students: $p<0.001$ (Fisher's exact test)

	Totally disagree	Disagree	Indifferent	Agree	Totally Agree	Total
Lecturers	2 (4.1%)	8 (16.3%)	2 (4.1%)	23 (46.9%)	14 (28.6%)	49 (100%)
Students	18 (8.1%)	75 (33.9%)	53 (24.0%)	58 (26.2%)	17 (7.7%)	221 (100%)

Lecturers X technical administrative: $p=0.491$ (Fisher's exact test)

Students X technical administrative: $p=0.019$ (Fisher's exact test)

	Totally disagree	Disagree	Indifferent	Agree	Totally Agree	Total
Students	18 (8.1%)	75 (33.9%)	53 (24.0%)	58 (26.2%)	17 (7.7%)	221 (100%)
Technical administrative	2 (10.5%)	5 (26.3%)	0 (0%)	9 (47.4%)	3 (15.8%)	19 (100%)

D) Years: $p<0.001$ (Chi-Square)

	Totally disagree	Disagree	Indifferent	Agree	Totally Agree	Total
First	16 (9.5%)	57 (33.9%)	48 (28.6%)	40 (23.8%)	7 (4.2%)	168 (100%)
Last	3 (3.8%)	22 (27.8%)	6 (7.6%)	33 (41.8%)	15 (19.0%)	79 (100%)

6 - At ESCS there is a methodological conflict in the teaching-learning process.

A) Absolute and Relative Frequencies

	Totally disagree	Disagree	Indifferent	Agree	Totally Agree	Total
Frequency	40 (13.8%)	114 (39.3%)	28 (9.7%)	81 (27.9%)	27 (9.3%)	290 (100%)

B) Course: $p=0.048$ (Chi-Square)

	Totally disagree	Disagree	Indifferent	Agree	Totally Agree	Total
Medicine	21 (14.7%)	67 (46.8%)	9 (6.3%)	34 (23.8%)	12 (8.4%)	143 (100%)
Nursing	19 (12.9%)	47 (32.0%)	19 (12.9%)	47 (32.0%)	15 (10.2%)	147 (100%)

C) Categories

Lecturers X Students: $p>0.05$ (Fisher's exact test)

Lecturers X Technical Administrative: $p>0.05$ (Fisher's exact test)

Students X Technical Administrative: p= 0.01 (Fisher's exact test)

	Totally disagree	Disagree	Indifferent	Agree	Totally Agree	Total
Students	37 (16.5%)	89 (39.7%)	21 (9.4%)	57 (25.4%)	20 (8.9%)	224 (100%)
Technical Administrative	0 (0%)	4 (21.1%)	5 (26.3%)	9 (47.4%)	1 (5.3%)	19 (100%)

D) Years: p<0.001 (Chi-Square)

	Totally disagree	Disagree	Indifferent	Agree	Totally Agree	Total
First	38 (22.1%)	76 (44.2%)	17 (9.9%)	34 (19.8%)	7 (4.1%)	172 (100%)
Last	0 (0%)	26 (32.9%)	6 (7.6%)	32 (40.5%)	15 (19.0%)	79 (100%)

7 - The Samambaia campus represents ESCS as much as the Plano Piloto campus

A) Absolute and relative frequencies

	Totally disagree	Disagree	Indifferent	Agree	Totally Agree	Total
Frequency	86 (29.5%)	73 (25.0%)	42 (14.4%)	55 (18.8%)	36 (12.3%)	292 (100%)

B) Course: p<0.001 (Chi-Square)

	Totally disagree	Disagree	Indifferent	Agree	Totally Agree	Total
Medicine	16 (11.2%)	36 (25.2%)	37 (25.9%)	38 (26.6%)	16 (11.2%)	143 (100%)
Nursing	70 (47.0%)	37 (24.8%)	5 (3.4%)	17 (11.4%)	20 (13.4%)	149 (100%)

C) Categories

Lecturers X Students: p>0.05 (Fisher's exact test)

Lecturers X Technical Administrative: p>0.05 (Fisher's exact test)

Students X Technical Administrative: p= 0.014 (Fisher's exact test)

	Totally disagree	Disagree	Indifferent	Agree	Totally Agree	Total
Students	74 (32.7%)	55 (24.3%)	32 (14.2%)	38 (16.8%)	27 (11.9%)	226 (100%)
Technical Administrative	1 (5.3%)	6 (31.6%)	1 (5.3%)	6 (31.6%)	5 (26.3%)	19 (100%)

D) Years: p= 0.026 (Chi-Square)

	Totally disagree	Disagree	Indifferent	Agree	Totally Agree	Total
First	43 (24.7%)	45 (25.9%)	29 (16.7%)	34 (19.5%)	23 (13.2%)	174 (100%)
Last	35 (44.3%)	18 (22.8%)	11 (13.9%)	10 (12.7%)	5 (6.3%)	79 (100%)

8 - The separation of medicine and nursing courses on two campuses makes organizational identity difficult

A) Absolute and relative frequencies

	Totally disagree	Disagree	Indifferent	Agree	Totally Agree	Total
Frequency	19 (6.5%)	44 (15.1%)	26 (8.9%)	92 (31.6%)	110 (37.8%)	291 (100%)

B) Course: $p<0.001$ (Chi-Square)

	Totally disagree	Disagree	Indifferent	Agree	Totally Agree	Total
Medicine	14 (9.9%)	31 (21.8%)	18 (12.7%)	42 (29.6%)	37 (26.0%)	142 (100%)
Nursing	5 (3.4%)	13 (8.7%)	8 (5.4%)	50 (33.5%)	73 (49.0%)	149 (100%)

C) Categories: $p> 0.05$ for all comparisons (there was no significant difference between lecturers, students or technical-administrative).

D) Years: $p<0.001$ (Chi-Square)

	Totally disagree	Disagree	Indifferent	Agree	Totally Agree	Total
First	17 (9.8%)	33 (19.1%)	21 (12.1%)	57 (32.9%)	45 (26.0%)	173 (100%)
Last	0 (0%)	5 (6.3%)	3 (3.8%)	23 (29.1%)	48 (60.8%)	79 (100%)

9 - The existence of ESCS has not been sufficient for its identification.

A) Absolute and Relative Frequencies

	Totally disagree	Disagree	Indifferent	Agree	Totally Agree	Total
Frequency	47 (16.2%)	123 (42.4%)	19 (6.6%)	82 (28.3%)	19 (6.6%)	290 (100%)

B) Course: $p=0.909$

C) Categories: $p>0.05$ for all comparisons (there was no significant difference between lecturers, students or technical-administrative).

D) Years: $p=0.087$

10 - With the creation of "Unisus", ESCS's social body will feel more strengthened in its identification with the institution

A) Absolute and relative frequencies

	Totally disagree	Disagree	Indifferent	Agree	Totally Agree	Total
Frequency	21 (7.2%)	37 (12.7%)	71 (24.4%)	118 (40.5%)	44 (15.1%)	291 (100%)

B) Course: $p<0.001$ (Chi-Square)

	Totally disagree	Disagree	Indifferent	Agree	Totally Agree	Total
Medicine	19 (13.3%)	27 (18.9%)	31 (21.7%)	55 (38.5%)	11 (7.7%)	143 (100%)
Nursing	2 (1.4%)	10 (6.8%)	40 (27.0%)	63 (42.6%)	33 (22.3%)	148 (100%)

C) Categories: p>0.05 for all comparisons (there was no significant difference between lecturers, students or technical-administrative).

D) Years: p=0.018 (Chi-Square)

	Totally disagree	Disagree	Indifferent	Agree	Totally Agree	Total
First	10 (5.8%)	14 (8.1%)	48 (27.7%)	73 (42.2%)	28 (16.2%)	173 (100%)
Last	10 (12.7%)	15 (19.0%)	18 (22.8%)	29 (36.7%)	7 (8.9%)	79 (100%)

11 - ESCS <u>lecturers</u> did not effectively incorporate the methodology adopted

A) Absolute and relative frequencies

	Totally disagree	Disagree	Indifferent	Agree	Totally Agree	Total
Frequency	67 (23.0%)	125 (43.0%)	26 (8.9%)	59 (20.3%)	14 (4.8%)	291 (100%)

B) Couse: p= 0.322 (Chi-Square)

C) Categories:

Lecturers X Students: p<0.001 (Fisher's exact test)

	Totally disagree	Disagree	Indifferent	Agree	Totally Agree	Total
Lecturers	1 (2.0%)	23 (46.9%)	5 (10.2%)	14 (28.6%)	6 (12.2%)	49 (100%)
Students	65 (28.8%)	93 (41.2%)	19 (8.4%)	41 (18.1%)	8 (3.5%)	226 (100%)

Lecturers X Technical Administrative: p=0.466 (Fisher's exact test)

Students X Technical Administrative: p=0.085 (Fisher's exact test)

D) Years: p<0.001 (Chi-Square)

	Totally disagree	Disagree	Indifferent	Agree	Totally Agree	Total
First	64 (37.0%)	79 (45.7%)	16 (9.2%)	14 (8.1%)	0 (0%)	173 (100%)
Last	2 (2.5%)	29 (36.7%)	5 (6.3%)	33 (41.8%)	10 (12.7%)	79 (100%)

12 - ESCS <u>students</u> did not effectively incorporate the methodology adopted

A) Absolute and relative frequencies

	Totally disagree	Disagree	Indifferent	Agree	Totally Agree	Total
Frequency	52 (17.9%)	162 (55.9%)	23 (7.9%)	46 (15.9%)	7 (2.4%)	290 (100%)

B) Course: p= 0.904 (Chi-Square)

C) Categories:

Lecturers X Students: p= 0.007 (Chi-Square)

	Totally disagree	Disagree	Indifferent	Agree	Totally Agree	Total
Lecturers	3 (6.0%)	30 (60.0%)	1 (2.0%)	14 (28.0%)	2 (4.0%)	50 (100%)
Students	49 (21.8%)	123 (54.7%)	19 (8.4%)	29 (12.9%)	5 (2.2%)	225 (100%)

Lecturers X Technical Administrative: p=0.219 (Fisher's exact test)

Students X Technical Administrative: p=0.074 (Fisher's exact test)

D) Years: p=0.021 (Chi-Square)

	Totally disagree	Disagree	Indifferent	Agree	Totally Agree	Total
First	38 (22.0%)	101 (58.4%)	15 (8.7%)	15 (8.7%)	4 (2.3%)	173 (100%)
Last	13 (16.5%)	41 (51.9%)	5 (6.3%)	19 (24.1%)	1 (1.3%)	79 (100%)

13 - There are several ESCS, including one real and the other ideal

A) Absolute and relative frequencies

	Totally disagree	Disagree	Indifferent	Agree	Totally Agree	Total
Frequency	12 (4.1%)	72 (24.8%)	44 (15.2%)	122 (42.1%)	40 (13.8%)	290 (100%)

B) Course: p = 0.216 (Chi-Square)

C) Categories:
Lecturers X Students: p=0.165 (Chi-Square)
Students X Technical Administrative: p=0.164 (Fisher's exact test)
Lecturers X Technical Administrative: p=0.035 (Fisher's exact test)

	Totally disagree	Disagree	Indifferent	Agree	Totally Agree	Total
Lecturers	2 (4.0%)	6 (12.0%)	6 (12.0%)	28 (56.0%)	8 (16.0%)	50 (100%)
Technical Administrative	0 (0%)	9 (47.4%)	1 (5.3%)	8 (42.1%)	1 (5.3%)	19 (100%)

D) Yeas: p<0.001 (Chi-Square)

	Totally disagree	Disagree	Indifferent	Agree	Totally Agree	Total
First	9 (5.2%)	57 (32.9%)	37 (21.4%)	63 (36.4%)	7 (4.1%)	173 (100%)
Last	1 (1.3%)	5 (6.4%)	3 (3.8%)	43 (55.1%)	26 (33.3%)	78 (100%)

14 - As there is no teaching career at the institution, the faculty does not feel as an integral part

A) Absolute and relative frequencies

	Totally disagree	Disagree	Indifferent	Agree	Totally Agree	Total
Frequency	54 (18.6%)	109 (37.5%)	52 (17.9%)	56 (19.2%)	20 (6.9%)	291 (100%)

B) Course: p= 0.248 (Chi-Square)

C) Categories

Lecturers X Students: $p<0.001$ (Chi-Square)

	Totally disagree	Disagree	Indifferent	Agree	Totally Agree	Total
Lecturers	5 (10.0%)	15 (30.0%)	1 (2.0%)	21 (42.0%)	8 (16%)	50 (100%)
Students	47 (20.8%)	89 (39.4%)	48 (21.2%)	30 (13.3%)	12 (5.3%)	226 (100%)

Lecturers X Technical Administrative: $p= 0.023$ (Fisher's exact test)

	Totally disagree	Disagree	Indifferent	Agree	Totally Agree	Total
Lecturers	5 (10.0%)	15 (30.0%)	1 (2.0%)	21 (42.0%)	8 (16%)	50 (100%)
Technical Administrative	3 (16.7%)	6 (33.3%)	4 (22.2%)	5 (27.8%)	0 (0%)	18 (100%)

Students X Technical Administrative: $p= 0.538$ (Fisher's exact test)

D) Years: $p<0.001$ (Chi-Square):

	Totally disagree	Disagree	Indifferent	Agree	Totally Agree	Total
First	45 (25.9%)	76 (43.7%)	32 (18.4%)	15 (8.6%)	6 (3.4%)	174 (100%)
Last	2 (2.5%)	24 (30.4%)	16 (20.3%)	26 (32.9%)	11 (13.9%)	79 (100%)
